# Cefiderocol Is Effective In Vitro Against Numerous Gram-Negative Species Isolated from Keratitis Patients

**DOI:** 10.3390/antibiotics15040348

**Published:** 2026-03-29

**Authors:** Jonathan B. Mandell, Robert M. Q. Shanks, Eric G. Romanowski

**Affiliations:** The Charles T. Campbell Ophthalmic Microbiology Laboratory, Department of Ophthalmology, University of Pittsburgh School of Medicine, Pittsburgh, PA 15219, USA; jbm42@pitt.edu (J.B.M.); shanksrm@upmc.edu (R.M.Q.S.)

**Keywords:** Gram-negative bacteria, keratitis, cefiderocol, antibiotic

## Abstract

Background: To evaluate the potential of cefiderocol as a topical ophthalmic antibiotic by determining the susceptibility of keratitis isolates from an extensive panel of Gram-negative bacterial species to this siderophore-cephalosporin class antibiotic. Methods: Minimum Inhibitory Concentrations (MICs) of cefiderocol were determined by the broth dilution method using iron-depleted, cation-adjusted Mueller–Hinton broth. The following Gram-negative bacteria were included: *Acinetobacter baumannii* (n = 13), *Achromobacter xylosoxidans* (n = 14), *Escherichia coli* (n = 15), *Klebsiella aerogenes* (n = 14), *Klebsiella pneumoniae* (n = 13), *Klebsiella oxytoca* (n = 14), *Moraxella* spp. (n = 15), *Proteus mirabilis* (n = 13), *Pseudomonas aeruginosa* (n = 17), *Serratia marcescens* (n = 14) and *Stenotrophomonas maltophilia* (n = 12). MIC_90_ values were calculated for each of the species. Results: MIC_90_ values (µg/mL): *A. baumannii* (0.5), *A. xylosoxidans* (0.25), *E. coli* (0.5), *K. aerogenes* (1.0), *K. oxytoca* (0.5), *K. pneumoniae* (0.5), *Moraxella* spp. (0.5), *P. mirabilis* (0.25), *P. aeruginosa* (0.5), *S. marcescens* (0.5), and *S. maltophilia* (0.25). In total, 100% of the isolates were determined to be susceptible to cefiderocol in vitro except for *A. xylosoxidans* and *Moraxella* spp., for which there are no established breakpoints for cefiderocol. Conclusions: Cefiderocol demonstrated in vitro activity against the tested panel of Gram-negative keratitis isolates. The results of this study suggest cefiderocol may be useful for the treatment of keratitis caused by numerous Gram-negative pathogens. Further development of cefiderocol for the topical treatment of Gram-negative keratitis is indicated.

## 1. Introduction

Bacteria are the most common cause of microbial keratitis, a vision-threatening disease. *Pseudomonas aeruginosa* (PA) is the most common Gram-negative pathogen responsible for bacterial keratitis, representing 18% of all bacterial keratitis cases seen in the western portion of Pennsylvania, USA, in our clinics. Other prevalent Gram-negative pathogens observed in bacterial keratitis cases include, but are not limited to, *Escherichia coli*, *Klebsiella* spp., *Moraxella* spp., and *Serratia marcescens* [1].

In general, topical antibiotic treatments such as fluoroquinolones (ciprofloxacin and moxifloxacin) and aminoglycosides (tobramycin) are successfully used to treat keratitis caused by Gram-negative bacteria [2]. But this might not always be the case. From 2022 to 2024, an outbreak of extensively drug-resistant (XDR) PA occurred in the USA that spread across 18 states, resulting in at least 81 cases of patients with positive cultures, four cases of enucleation due to ocular infections, and four deaths [3,4,5,6]. Epidemiological studies traced the outbreak of infections to the use of over-the-counter artificial tear eye drops [3,4,5,6]. In addition to the artificial tears outbreak, another study investigated two XDRPA infections in canines in which the XDRPA isolates were genetically similar to those from the human artificial tear outbreak [7].

Isolates from multiple patients were obtained by the Center for Disease Control, including one from a keratitis patient, CDC1270. This XDRPA isolate was found to be resistant to almost all antibiotics tested across multiple classes, including those routinely used for Gram-negative bacterial keratitis, including ceftazidime, tobramycin, and ciprofloxacin [5,8]. This XDRPA was found to be of a sequence type (ST1203) never seen before in the US and notable for Guiana extended-spectrum as well as Verona metallo-β-lactamase expression [9,10]. Interestingly, CDC1270 was found to be susceptible to a member of a new class of β-lactam antibiotics known as siderophore-cephalosporins. That antibiotic was cefiderocol.

Cefiderocol was approved by the FDA in 2019 and 2020 for the treatment of complicated urinary tract infections and nosocomial bacterial pneumonia [11]. It has displayed excellent in vitro activity against Gram-negative bacteria [12,13,14], including multidrug-resistant isolates [15,16,17,18]. Cefiderocol was rationally designed by the incorporation of side chains of previous cephalosporin drugs, which increase its tolerance to β-lactamase activity [19,20,21]. In particular, cefiderocol incorporates a pyrrolidinium group on the C-3 side chain (similar to cefepime), which improves antibacterial activity and stability against β-lactamases [21]. Furthermore, cefiderocol also incorporates a carboxypropanoxyimino group on the C-7 side chain (similar to ceftazidime) that improves its transport across the bacterial outer membrane [21].

Cefiderocol contains a novel chlorocatechol group on the C-3 side chain that confers siderophore activity [21]. This chlorocatechol group captures iron from the environment and then binds to a bacterial outer membrane iron transport protein, after which the entire antibiotic molecule is actively transported into the periplasmic space of the bacterial cell [21]. The chlorocatechol group dissociates from the molecule, and cefiderocol thereby exerts its antibacterial effects by targeting the penicillin-binding protein 3 (PBP3) and inhibiting peptidoglycan synthesis [21]. This uptake mechanism is called a “Trojan Horse” strategy, as cefiderocol “tricks” the bacteria into uptaking the antibiotic [21].

Cefiderocol is administered by intravenous infusion. It was not used topically to treat the XDRPA keratitis during the artificial tears outbreak, and many in the ophthalmic community asked “why not” since other cephalosporin antibiotics (ceftazidime, cefazolin) are prepared as fortified antibiotics for use in the treatment of bacterial keratitis. Unfortunately, at that time, the safety and efficacy of cefiderocol had never been evaluated for topical ocular use. Only recently was cefiderocol experimentally evaluated for use in the eye and for the topical treatment of XDRPA keratitis in animal models [22,23]. Until our recent studies [22,23], it was unknown whether cefiderocol would be safe for topical ocular use, whether it would be efficacious in the treatment of XDRPA bacterial keratitis, whether it would penetrate the corneal epithelium to the corneal stroma, or whether it would be stable enough to be used topically for several days due to the warnings contained in the cefiderocol (Fetroja^®^, Shionogi, Inc.) package insert. In our first study, we determined that cefiderocol, at a concentration of 50 mg/mL, was stable (maintaining antimicrobial activity for up to a month when refrigerated and kept out of light) when formulated in saline, was non-toxic to the ocular surface after topical dosing, and was effective in vivo in reducing XDRPA bacterial corneal colony counts after topical treatment in a rabbit model with the corneal epithelium removed [22]. We subsequently showed an important pharmacodynamic property of topical cefiderocol in that the efficacy of topical 50 mg/mL cefiderocol was dependent on the state of the corneal epithelium of treated animal eyes, with antibacterial efficacy being reduced when the corneal epithelium was mostly intact [23]. This suggests that cefiderocol does not penetrate the corneal epithelium as efficiently as it does when the corneal epithelium is removed, as would occur in a corneal ulcer [23].

We also pondered whether cefiderocol could be used as an empiric treatment for bacterial endophthalmitis. With the rise in antibiotic resistance among Gram-negative bacteria and the common use of ceftazidime as an empiric endophthalmitis treatment, could cefiderocol be used as an alternative? While there have been no studies evaluating the intraocular safety and efficacy of cefiderocol, we have demonstrated that a panel of Gram-negative endophthalmitis isolates was 100% susceptible to cefiderocol in vitro [24].

Given all these promising results against XDRPA and Gram-negative endophthalmitis isolates, further investigation is warranted to verify the utility of cefiderocol for coverage of other Gram-negative keratitis pathogens. As a next step to understanding the potential of cefiderocol for treating Gram-negative bacterial keratitis, the antibacterial activity of cefiderocol was evaluated in vitro against an extensive panel of the most common Gram-negative corneal pathogens isolated from keratitis patients.

## 2. Results

A total of 153 Gram-negative bacterial keratitis isolates were evaluated in this study. We reviewed the antibiotic susceptibility results for those 153 isolates to determine whether any of the isolates were considered multidrug resistant (MDR). A bacterial isolate is considered MDR if it is resistant to at least 1 antibiotic from at least 3 different antimicrobial classes [25]. Our review determined that none of the isolates tested were considered MDR based on the three classes of antibiotics (fluoroquinolones, aminoglycosides, and polymyxins) that are used in our laboratory for routine antibiotic susceptibility testing (AST) of Gram-negative bacteria. Since topical antibiotic therapy for keratitis is limited to a few agents and classes, only targeted AST to those agents is performed in our laboratory using selected antibiotics among the three classes. Ciprofloxacin was the representative of the fluoroquinolones, tobramycin was the representative of the aminoglycosides, and polymyxin B was the representative of the polymyxins. Therefore, we may be underreporting the instances of MDR isolates in our study population due to the lack of several representatives for each antibiotic class.

The breakdown of Gram-negative bacterial species and the number of isolates used from each species in this study are listed in Table 1 along with the reviewed antibiotic susceptibility data.

Among the isolates, for the three tested antibiotics, *A. baumannii*, *K. aerogenes*, *K. oxytoca*, *K. pneumoniae*, *Moraxella* spp., and *P. aeruginosa* demonstrated excellent susceptibility to all three antibiotics. The isolates of *P. mirabilis* and *S. marcescens* demonstrated susceptibility to ciprofloxacin and tobramycin but demonstrated intrinsic resistance to polymyxin B [26]. The *E. coli* isolates demonstrated some resistance to ciprofloxacin and tobramycin but were 100% susceptible to polymyxin B. The isolates of *A. xylosoxidans* were mostly susceptible to ciprofloxacin but, in contrast, were mostly resistant to tobramycin. However, similar to *E. coli*, the *A. xylosoxidans* isolates demonstrated 100% susceptibility to polymyxin B. Finally, the *S. maltophilia* isolates demonstrated some resistance among all three antibiotics tested, but each isolate was susceptible to at least one of the other antibiotics tested.

The minimum inhibitory concentrations (MICs) for all isolates are presented in Supplementary Table S1. Table 2 summarizes these results by presenting the median MICs, mode MICs, MIC_90_s, range of MICs, and susceptibility data based on experiments using the broth dilution MIC method. As there are no susceptibility breakpoints for topical or intraocular therapy of the eye, systemic cefiderocol Clinical Laboratory Standards Institute (CLSI) MIC breakpoints were used for our analysis. The highest MIC for any individual bacterium was 2 µg/mL for a single keratitis isolate of *P. aeruginosa*. For *Pseudomonadales* (*P. aeruginosa*, *A. baumannii*, and *Moraxella* spp.) and *Enterobacterales* (*S. marcescens*, *P*. *mirabilis*, *E. coli*, *K. aerogenes*, *K. oxytoca*, and *K. pneumoniae*), the susceptibility breakpoint for cefiderocol is ≤4 µg/mL. For *S. maltophilia*, the susceptibility breakpoint of cefiderocol is ≤1 µg/mL. There are no CLSI cefiderocol susceptibility breakpoints for *Moraxella* spp. and *A. xylosoxidans.* For these species, we chose the more stringent of the CLSI breakpoints, ≤1 µg/mL, to determine susceptibility. Based on these criteria, 100% of the Gram-negative isolates in the panel evaluated demonstrated susceptibility to cefiderocol (Table 1).

The MIC of the XDRPA CDC1270 outbreak isolate was determined to be 0.125 µg/mL. This is the same MIC that was determined using the Epsilometer strip testing that was reported in our previous study [22]. The MICs for the American Type Culture Collection (ATCC) quality control strains were evaluated in each trial and produced MICs within the acceptable range of 0.06–0.5 µg/mL, thus validating the concentrations of cefiderocol.

## 3. Discussion

Infectious keratitis is routinely caused by a wide variety of microbial agents, including bacteria (Gram-positive and Gram-negative), fungi (yeast and mold), viruses (herpesvirus), and parasites (acanthamoeba) [1]. Bacterial keratitis accounts for the majority of infectious keratitis [1]. The recent outbreak of XDRPA infections associated with the use of over-the-counter, unpreserved, multi-dose artificial tears highlights the need for new antibiotic treatments for challenging ocular infections. This is especially true for hard-to-treat Gram-negative keratitis. Keratitis caused by Gram-positive pathogens can be successfully treated with vancomycin, to which little to no resistance has been demonstrated [27].

Multidrug-resistant Gram-negative bacteria have displayed a worrying increase in antibiotic resistance over the past decades [28]. Due to the paucity of new antibiotic treatments, the World Health Organization classified several of these Gram-negative bacterial species as priority pathogens in 2017 [28]. These are known as ESKAPE pathogens (*Enterococcus faecium*, *Staphylococcus aureus*, *Klebsiella pneumoniae*, *Acinetobacter baumannii*, *Pseudomonas aeruginosa*, and *Enterobacter* species) [29]. These pathogens represent a global threat to human health. This classification emphasizes the urgent need for research and development of novel antibiotic therapies.

Antibiotic resistance mechanisms can be broadly categorized into four groups: (1) inactivation or alteration of the antimicrobial molecule (e.g., β-lactamases); (2) bacterial antibiotic target site modifications (e.g., LPS modification and PBP2a expression with reduced β-lactam affinity); (3) reduced antibiotic penetration/accumulation (e.g., efflux pumps); and (4) the formation of bacterial biofilms, which increase bacterial tolerance to antibiotics [29]. The antibiotic cefiderocol specifically targets the first and third categories listed above. It has a structure like that of cefepime (pyrrolidinium group on the C-3 side chain, which improves stability against β-lactamases) and ceftazidime (same C-7 side chain conferring improved stability against β-lactamases) [30]. Cefiderocol also contains an iron-binding siderophore group conjugated to a β-lactam group that hijacks the iron uptake systems of Gram-negative bacteria and therefore circumvents the outer membrane barriers and efflux pumps [15,30]. The increased accumulation of cefiderocol in the bacterial cells and its stability against β-lactamases result in increased antibacterial activity.

We have previously reported that cefiderocol demonstrated excellent antibacterial activity against 135 *P. aeruginosa* keratitis isolates, producing an MIC_90_ of 0.125 µg/mL and 100% susceptibility using epsilometer testing [22]. However, while epsilometer testing is validated for testing cefiderocol against *P. aeruginosa*, one report voiced concerns that epsilometer testing of cefiderocol could be inaccurate due to potentially higher concentrations of iron in standard Mueller–Hinton II agar plates [31]. Therefore, we wished to confirm the antibacterial activity of cefiderocol against additional *P. aeruginosa* keratitis isolates using broth dilution MIC testing in iron-depleted, cation-adjusted Mueller–Hinton broth (ID-CA-MHB), the reference method for MIC testing of cefiderocol. We also wanted to evaluate the antibacterial activity of cefiderocol against additional species of Gram-negative bacteria isolated from corneal infections. In the current study, we confirmed the excellent antibacterial activity of cefiderocol against an additional 16 *P. aeruginosa* keratitis isolates, producing an MIC_90_ of 0.5 µg/mL and 100% susceptibility using broth dilution MIC testing in ID-CA-MHB. In addition, the MIC produced by the 2023 XDR *P. aeruginosa* CDC1270 strain by broth dilution MIC testing, 0.125 µg/mL, was the same as that produced using epsilometer testing. In addition, the MICs produced by broth dilution MIC testing in ID-CA-MHB for the ATCC quality control reference strains, *P. aeruginosa* ATCC 27853 and *E. coli* ATCC 25922, were within the acceptable MIC range, thus validating the cefiderocol concentrations and the study.

The cefiderocol MICs produced for common Gram-negative keratitis isolates of *Achromobacter xylosoxidans*, *Escherichia coli*, *Klebsiella oxytoca*, *Moraxella* spp., *Proteus mirabilis*, *Serratia marcescens*, *Stenotrophomonas maltophilia*, and the additional ESKAPE pathogens *Klebsiella pneumoniae*, *Acinetobacter baumannii*, and *Klebsiella (Enterobacter) aerogenes* were all ≤1 µg/mL and were 100% susceptible based on our listed criteria. These results suggest that topical cefiderocol may be effective in the topical treatment of keratitis caused by these pathogens, should first-line therapy be ineffective.

Limitations of this study are that none of the keratitis isolates evaluated across all species were multidrug-resistant or extensively drug-resistant. Fortunately, these are exceedingly rare among those bacteria causing keratitis at our vision institute. However, when we encounter multidrug-resistant or extensively drug-resistant keratitis isolates of these Gram-negative pathogens that include resistance to the antibiotics commonly used to treat bacterial keratitis, we now have evidence that cefiderocol could be used off-label to topically treat these infections. However, topical cefiderocol has yet to be used clinically in keratitis patients; therefore, it is possible that the in vitro susceptibility data against these Gram-negative pathogens may not translate to clinical efficacy. We additionally have not determined the specific mechanism by which cefiderocol targets each bacterium beyond what is already known in the field [21].

To potentially improve the efficacy of cefiderocol and other antibiotics as topical treatments for keratitis, we have recently reported that in vitro combinations of cefiderocol with the common keratitis treatments of moxifloxacin or polymyxin B produced synergistic interactions against *Pseudomonas aeruginosa* keratitis isolates [32,33].

## 4. Materials and Methods

### 4.1. Bacterial Strains

De-identified, laboratory-confirmed, Gram-negative bacterial strains isolated from patients with keratitis presenting to the Department of Ophthalmology at the University of Pittsburgh School of Medicine, in Pittsburgh, PA, USA, were used to determine MICs for cefiderocol. Standard biochemical methods or MALDI-TOF mass spectroscopy (Beckman Coulter, Brea, CA, USA) were used to identify the bacteria. Following identification and antibiotic susceptibility testing for patient treatment, the bacterial isolates were frozen at −80 °C in 15% glycerol. The bacteria evaluated in this study were all keratitis isolates, listed in Table 1. The CDC1270 XDRPA isolate was also included among the isolates tested. ATCC quality control bacteria (*P. aeruginosa* ATCC 27853 and *E. coli* ATCC 25922) were used in each trial. Routine susceptibility testing of the keratitis isolates using ciprofloxacin, tobramycin, and polymyxin B was conducted at the time of isolation using either Kirby-Bauer disk diffusion or MIC strip testing [34]. Susceptibility was determined based on the CLSI systemic breakpoints [34]. There are no susceptibility breakpoints for topical ocular therapy.

### 4.2. Cefiderocol

One-gram vials of cefiderocol were provided by Shionogi, Inc., Florham Park, NJ, USA. The vials were stored at 4 °C until reconstituted with 10 mL of sterile saline according to the manufacturer’s instructions. The reconstituted solution had a final volume of approximately 11.0 mL and a concentration of 90.9 mg/mL. Then, 2× concentrations (32, 16, 8, 4, 2, 1, 0.5, 0.25, 0.125, 0.0625, and 0.03125 µg/mL) of cefiderocol were prepared in ID-CA-MHB. Once the dilutions were completed, 100 µL of each 2× antibiotic concentration was added in columns 1–11 of 96-well polystyrene, round-bottom plates (Falcon 351177, ThermoFisher Scientific, Pittsburgh, PA, USA). The highest concentration is in column 1, and the lowest concentration is in column 11. ID-CA-MHB without antibiotic was added to column 12. The plates were wrapped in parafilm and frozen at −80 °C until use.

### 4.3. Antibacterial Testing

Cefiderocol MIC values were determined using a modified CLSI broth dilution methodology incorporating the use of ID-CA-MHB (ThermoFisher Scientific, Pittsburgh, PA, USA [Specialty Microbiology Products Custom Prepared Media: T3464-10]). Briefly, the bacterial keratitis isolates and ATCC quality control isolates (*P. aeruginosa* ATCC 27853 and *E. coli* ATCC 25922) were thawed and grown on trypticase soy agar with 5% sheep erythrocytes. After overnight growth, several colonies from each bacterial isolate were suspended in 2 mL of PBS and adjusted to a 0.5 McFarland standard (~1.2 × 10^8^ CFU/mL). Twenty microliters (20 µL) of the bacterial suspensions were added to 2 mL of ID-CA-MHB to produce a bacterial inoculum of approximately 5 × 10^5^–1 × 10^6^ CFU/mL. One hundred microliters (100 µL) of each bacterial suspension was added to one row of the thawed and warmed cefiderocol 96-well plates to yield final test concentrations of 16, 8, 4, 2, 1, 0.5, 0.25, 0.125, 0.0625, 0.03125, 0.0156, and 0.0 µg/mL. Once the plate was inoculated with 8 different bacterial isolates, the plates were placed on a shaker for 15 min at room temperature. After shaking, the plates were incubated overnight in an air incubator at 37 °C. The following day, the MICs were determined visually as the lowest concentration of drug that inhibited visible bacterial growth according to the CLSI criteria established for cefiderocol [35]. MICs for the quality control ATCC isolates for cefiderocol must be within the acceptable range from 0.06 to 0.5 µg/mL to validate the concentrations of cefiderocol used in the study, thus validating the experimental results. A consensus among 3 evaluators (J.B.M., R.M.Q.S., and E.G.R.) determined the final MICs.

### 4.4. Statistical Analysis

Statistical analysis of MIC data (median, mode, MIC_90_, range of MICs) was performed using Minitab Version 19 (Minitab, State College, PA, USA) statistical software.

## 5. Conclusions

The results of this in vitro study provide evidence that fortified cefiderocol could be a future treatment for keratitis caused by Gram-negative bacteria. We have previously demonstrated that topical fortified cefiderocol was safe and effective in the treatment of experimental keratitis caused by extensively drug-resistant *P. aeruginosa* [22,23]. Only human clinical trials can definitively determine whether fortified cefiderocol demonstrates clinical effectiveness in the treatment of Gram-negative bacterial keratitis. Further investigation of cefiderocol for the topical treatment of Gram-negative keratitis is warranted.

## Figures and Tables

**Table 1:** Gram-negative keratitis isolates and their percent susceptibilities to ciprofloxacin, tobramycin, and polymyxin B.

Bacteria	n	Fluoroquinolones	Aminoglycosides	Polymyxins
		(Ciprofloxacin)	(Tobramycin)	(Polymyxin B)
*A. xylosoxidans*	14	13/14 (92.9%)	1/14 (7.1%)	14/14 (100%)
*A. baumannii*	13	13/13 (100%)	13/13 (100%)	13/13 (100%)
*E. coli*	15	12/15 (80%)	7/15 (46.7%)	15/15 (100%)
*K. aerogenes*	14	14/14 (100%)	14/14 (100%)	13/14 (92.9%)
*K. oxytoca*	14	13/14 (92.9%)	14/14 (100%)	14/14 (100%)
*K. pneumoniae*	13	13/13 (100%)	13/13 (100%)	13/13 (100%)
*Moraxella* spp.	15	15/15 (100%)	15/15 (100%)	15/15 (100%)
*P. mirabilis*	13	11/13 (84.6%)	13/13 (100%)	0/13 (0%)
*P. aeruginosa*	16	16/16 (100%)	16/16 (100%)	16/16 (100%)
*S. marcescens*	14	14/14 (100%)	14/14 (100%)	5/13 (35.7%)
*S. maltophilia*	12	10/12 (83.3%)	6/12 (50%)	10/12 (83.3%)

**Table 2:** Gram-negative keratitis isolate MIC (µg/mL) and susceptibility to cefiderocol.

Bacteria	n	Median MIC	Mode MIC	MIC_90_	Range	Susceptibility (%)
*A. baumannii*	13	0.125	0.125, 0.25, 0.5	0.25	0.03125–1.0	13/13 (100%)
*E. coli*	15	0.125	0.125	0.5	0.125–1.0	15/15 (100%)
*K. aerogenes*	14	0.5	0.5	1.0	0.25–1.0	14/14 (100%)
*K. oxytoca*	14	0.25	0.125, 0.25	0.5	0.0625–0.5	14/14 (100%)
*K. pneumoniae*	13	0.125	0.25	0.5	0.0156–0.5	13/13 (100%)
*P. mirabilis*	13	0.03125	0.03125	0.25	0.03125–0.25	13/13 (100%)
*P. aeruginosa*	16	0.25	0.25	0.5	0.03125–2.0	16/16 (100%)
*S. marcescens*	14	0.25	0.125, 0.25	0.5	0.125–0.5	14/14 (100%)
*S. maltophilia*	12	0.0938	0.0625	0.25	0.03125–1.0	12/12 (100%)
*A. xylosoxidans*	14	0.0938	0.03125	0.25	0.03125–0.5	14/14 (100%) *
*Moraxella* spp.	15	0.125	0.25	0.5	0.0156–0.5	15/15 (100%) *

* There are no susceptibility breakpoints for cefiderocol to *A. xylosoxidans* and *Moraxella* spp. For these species, we chose the more stringent of the CLSI breakpoints, ≤1 µg/mL, to determine susceptibility.

## Data Availability

The data reported in this study are available in this manuscript.

## Supplementary Materials

The following supporting information can be downloaded at https://www.mdpi.com/article/10.3390/antibiotics15040348/s1. Table S1. Cefiderocol MICs for Gram-negative Keratitis Isolates in µg/mL.

## Abbreviations

The following abbreviations are used in this manuscript:

PA	*Pseudomonas aeruginosa*
XDR	Extensively drug-resistant
MIC	Minimum inhibitory concentration
ATCC	American Type Culture Collection
ID-CA-MHB	Iron-depleted, cation-adjusted, Mueller–Hinton broth
CLSI	Clinical Laboratory Standards Institute
µg/mL	Micrograms per milliliter
LPS	Lipopolysaccharide
PBP2a	Penicillin-binding protein 2a
mg/mL	Milligrams per milliliter
spp.	Species
MIC_90_	Minimum inhibitory concentration that inhibits the growth of 90% of strains tested
CFU/mL	Colony-forming units per milliliter
