# Supplementary material for: Cefiderocol Is Effective In Vitro Against Numerous Gram-Negative Species Isolated from Keratitis Patients
Source: Antibiotics (Basel). 2026 Mar 29;15(4):348. doi: 10.3390/antibiotics15040348 (PMC13113829; doi:10.3390/antibiotics15040348)
Supplement: Supplementary file 1 [file antibiotics-15-00348-s001.zip › antibiotics-4179927-supplementary.pdf]

Table S1. Cefiderocol MICs for Gram-negative Keratitis Isolates in μg/mL.

	A. baumannii		*E. coli*		*K. aerogenes*		*K. oxytoca*		*K. pneumoniae*		*P. mirabilis*		*P. aeruginosa*		*S. marcescens*		*S. maltophilia*		*A. xylosoxidans*		*Moraxella spp.*	
	Isolate	MIC	Isolate	MIC	Isolate	MIC	Isolate	MIC	Isolate	MIC	Isolate	MIC	Isolate	MIC	Isolate	MIC	Isolate	MIC	Isolate	MIC	Isolate	MIC
1.	K3371	0.03125	K3431	0.125	K3439	0.25	K3162	0.5	K3409	0.125	K3276	0.25	K3454	0.5	K3421	0.25	K3446	0.0625	K3145	0.125	K3447	0.25
2.	K2563	0.125	K3347	0.125	K3056	0.5	K3133	0.25	K3275	0.25	K3272	0.03125	K3437	0.125	K3418	0.5	K3085	0.0625	K3054	0.25	K3427	0.125
3.	K2158	0.3125	K3273	0.0156	K2840	0.5	K3063	0.125	K3071	0.125	K3152	0.03125	K3433	0.25	K3240	0.125	K2933	0.0625	K2923	0.03125	K3412	0.0625
4.	K1971	1	K2872	0.0156	K2550	0.25	K3050	0.125	K2829	0.5	K3144	0.0156	K3429	0.03125	K3236	0.25	K2925	0.125	K2917	0.0625	K3374	0.125
5.	K1880	0.125	K2753	0.5	K2103	0.5	K2991	0.5	K2477	0.0625	K3017	0.0156	K3424	0.25	K3165	0.5	K2834	0.25	K2871	0.0625	K3364	0.0625
6.	K1402A	0.25	K2641	0.25	K1686	0.5	K2857	0.125	K2372	0.25	K2837	0.03125	K3423	0.5	K3146	0.25	K2759	0.25	K2848	0.125	K3330	0.0156
7.	K1401	0.5	K2554	0.125	K1543	0.5	K2763	0.25	K1717	0.25	K2694	0.03125	K3415	0.125	K3126	0.5	K2613	0.0625	K2608	0.03125	K3328	0.125
8.	K1395	0.5	K2517	0.5	K1488	1	K2575	0.0625	K1660	0.25	K2676	0.25	K3404	2	K3097	0.125	K2509	1	K2605	0.03125	K3325	0.5
9.	K1363B	0.125	K1671	1	K1426	0.25	K2197	0.125	K1602	0.0625	K2675	0.03125	K3399	0.25	K3075	0.25	K2443	0.0625	K2604	0.03125	K3324	0.0156
10.	K1309	0.5	K1565	0.03125	K1297	1	K2157	0.125	K959	0.5	K2514	0.0625	K3398	0.25	K3068	0.5	K2437	0.25	K2441	0.5	K3314	0.125
11.	K1280	0.25	K1359	0.25	K1221	0.5	K2130	0.25	K733	0.125	K2454	0.125	K3394	0.5	K3045	0.125	K2365	0.125	K2438	0.03125	K3253	0.25
12.	K1101	0.0625	K877	0.25	K1175	0.5	K2097	0.25	K650	0.03125	K2345	0.03125	K3390	0.25	K2799	0.125	K2318	0.03125	K2373	0.125	K3252	0.0156
13.	K1100	0.25	K805	0.25	K1170	1	K2092	0.25	K387	0.0156	K2128	0.0126	K3386	0.125	K2662	0.125			K2254	0.125	K3246	0.0156
14.			K746	0.125	K1054	0.25	K2087	0.5					K2926	0.5	K2583	0.5			K1919	0.25	K3241	0.25
15.			K694	0.125									K1706	0.25							K2318	0.125
16.													K2259	0.125								
